# Road Traffic Noise, Obesity, and the Risk of Incident Type 2 Diabetes: A Cohort Study in UK Biobank

**DOI:** 10.3389/ijph.2022.1605256

**Published:** 2022-10-12

**Authors:** Lei Zuo, Xia Chen, Mingliang Liu, Li Chen, Wenbin Xu, Haiyan Chen, Shan Dong, Yuan Wei, Liangming Li, Shuang Peng, Guang Hao

**Affiliations:** ^1^ Department of Public Health and Preventive Medicine, School of Medicine, Jinan University, Guangzhou, China; ^2^ Department of Medicine, Georgia Prevention Institute, Medical College of Georgia, Augusta University, Augusta, GA, United States; ^3^ School of Environmental Science and Engineering, Guangdong University of Technology, Guangzhou, Guangdong, China; ^4^ Department of Parasitic Disease and Endemic Disease Control and Prevention, Guangzhou Center for Disease Control and Prevention, Guangzhou, China; ^5^ Guangzhou First People’s Hospital, The Second Affiliated Hospital of South China University of Technology, Guangzhou, China; ^6^ Key Laboratory of Sports Technique, Tactics and Physical Function of General Administration of Sport of China, Scientific Research Center, Guangzhou Sport University, Guangzhou, China; ^7^ School of Sport and Health Sciences, Guangzhou Sport University, Guangzhou, China; ^8^ Guangdong Key Laboratory of Environmental Exposure and Health, Jinan University, Guangzhou, China

**Keywords:** cohort study, obesity, interaction, noise, T2DM

## Abstract

**Objectives:** To assess the association of road traffic noise exposure with Type 2 Diabetes (T2D) risk, and to explore the potential moderation effect of obesity.

**Methods:** A total of 305,969 participants from the UK Biobank Cohort - an open access cohort of 500,000 participants recruited in the United Kingdom (UK) between 2006 and 2010 - were included in the study. A Cox proportional hazard model was fitted to assess the association between road traffic noise exposure and T2D.

**Results:** A total of 19,303 participants were diagnosed with T2D during the 11.9-year median follow-up period. For every 10 dB increase in road traffic noise, there was a 4% increase in T2D risk (HR = 1.04, 95%CI: 1.01, 1.07). Besides, a significant positive interaction was observed between obesity and road traffic noise (*P* interaction <0.001) for the risk of T2D. The association of road traffic noise with T2D was stronger in overweight and obese participants (HR = 1.04, 95% CI: 1.01–1.08), but not significant among lean ones (HR = 0.96, 95% CI: 0.86–1.07).

**Conclusion:** Our study observed a longitudinal association of road traffic noise exposure with T2D risk, which was stronger among overweight and obese individuals than the lean ones.

## Introduction

Type 2 diabetes (T2D), a chronic disease characterized by abnormal blood glucose levels and insulin resistance [1], has become a global public health issue. It is estimated that in 2019, a total of 463 million adults suffered from diabetes worldwide, and approximately 90%–95% of them were T2D, which will amount to 700 million by 2045 [2]. One-eighth of the world’s adults will die from diabetes and its complications in 2045 [3]. The latest edition of the Diabetes Map published by the International Diabetes Federation (IDF) shows that, in 2019, the global direct cost triggered by diabetes is estimated to be $760 billion, which will rise to $845 billion by 2045 [4].

A high-calorie diet [5, 6], low physical activity [7, 8], and unhealthy sleep patterns are recognized as traditional risk factors for T2D [9, 10]. However, these known risk factors are far from fully explaining the variations in T2D risk [11]. Epidemiological evidence indicates that noise exposure might be an underestimated risk factor for T2D, especially road traffic noise [12–14]. Several cohort studies have reported a significant positive association between road traffic noise and T2D [13–16], but the results of recent large-scale cohort studies remain inconsistent, which may be attributed to differences in the study population, noise estimation methods, and confounding adjustment [13, 14, 17, 18]. On the other hand, studies have shown that obesity might amplify the detrimental effects of noise exposure on human health in various organ systems [19, 20]. However, the effect modification of obesity on the relationship between road noise exposure and T2D has never been studied.

In this study, we used prospective cohort data from the UK Biobank to investigate the relationship between road traffic noise and T2D risk. In addition, we explored the potential effect modification of obesity on this association.

## Methods

### Study Population

This research has been carried out using the UK Biobank data through Application Number 69597 (https://www.ukbiobank.ac.uk/enable-your-research/approved-research). The primary aims of the project are to study the associations between noise pollution and cardiometabolic risk, and the roles of mental health and sleep quality in those associations. The UK Biobank is a large open access prospective cohort study aimed at improving the health status and prevention, diagnosis, and treatment of diseases in the United Kingdom. More than 500,000 participants aged 40–70 years were recruited from 2006 to 2010. Extensive baseline health information of participants was collected by a touch-screen questionnaire. Anthropometric and biometric measurements were also taken. Details of the study design can be found elsewhere [21]. All participants provided written consent, and ethical approval was obtained from the North West Multi-Centre Research Ethics Committee (London, U.K.). After excluding 81,111 participants with T2D and missing data on road traffic noise exposure at baseline, 421,302 participants remained in the cohort. We further excluded the ones who lacked data on age, body mass index (BMI), race, physical activity, smoking, alcohol consumption, educational attainment, sleep quality, and fruit/vegetable intake. Finally, 305,969 participants were included in the primary analysis.

### Road Traffic Noise Exposure Assessment

The annual average road traffic noise level at the address level was estimated based on the simplified version of the CNOSSOS-EU noise modeling framework [22, 23]. Moreover, the model has been adopted in epidemiological analyses, with a relatively good performance for exposure ranking (Spearman’s rank = 0.75) [24, 25].

The annual mean A-weighted sound pressure level in decibels (dB[A]) for 2009 was estimated on all roads within 500 m of participants’ home addresses at baseline. The model takes into account detailed information on noise propagation (refraction and diffraction), buildings absorption and land use, land cover, road network geography, meteorology, the distance between receivers and source and visual angle, building height, and calculated hourly vehicle flows using daily average traffic profile. The weighted 24-h average noise (Lden) and nighttime noise (23:00 to 07:00, Lnight) levels were estimated respectively. Given their high correlation, the average 24-hour noise level was used in our main analyses.

### Covariates

Age, sex, race, educational attainment, smoking, alcohol consumption, vegetable, and fruit intake, mental health, and sleep quality were collected using a touch-screen questionnaire. BMI was calculated by dividing weight (kg) by height (m^2^). Metabolic equivalent (MET) was computed in the light of the International Physical Activity Questionnaire [26]. Smoking and drinking status were classified into three groups (current, past, and never). Educational attainment was estimated according to the International Standard Education Classification (ISCED) as follows: none of the above (no qualifications) = 7 years of education; CSEs or equivalent = 10 years; O levels/GCSEs or equivalent = 10 years; A levels/AS levels or equivalent = 13 years; other professional qualification = 15 years; NVQ or HNC or equivalent = 19 years; college or university degree = 20 years of education. The highest classification was assigned to respondents who selected multiple options [27]. Symptoms of nerves, anxiety, nervousness or depression [NATD], major depression, and bipolar disorder were evaluated by an effective questionnaire [28] and defined as mental health (had NATD, major depression, and bipolar disorder vs. without these diseases) [29]. Please refer to Supplementary Methods for more details. A healthy sleep score (0–4) was calculated using sleep duration, insomnia, snoring, and nap during the day collected using a touch-screen questionnaire [30]. The participants with higher scores had a healthier sleeping pattern (more details in Supplementary Methods) [31]. The land-use regression model developed by the European Air Pollution Impact Cohort Study (ESCAPE) project was adopted to estimate the annual mean PM_2.5_ of home addresses in 2010 [32, 33].

### Assessment of Health Outcomes

Admissions and diagnoses data of hospital inpatient records obtained from the Hospital Episode Statistics for England, Scottish Morbidity Record data for Scotland, and the Patient Episode Database for Wales were used to ascertain T2D by the ICD-10 code of E11 [34]. Detailed information regarding the T2D definition is provided on the website of UK Biobank (https://biobank.ctsu.ox.ac.uk/showcase/label.cgi?id=2000).

### Statistical Analysis

Continuous variables were represented by mean and standard deviation, and categorical variables were described by cases (n) and percentages (%). The COX proportional hazard model was used to examine the associations of road traffic noise exposure with T2D. A directed acyclic graph (DAG) graph (Supplementary Figure S1) was plotted to identify confounding factors [35]. Model 1 were unadjusted for any covariates. Model 2 was adjusted for age, gender, ethnicity, physical activity, and educational attainment. Multipollutant model additionally included PM_2.5_ (Primary model). Model 3 was adjusted for smoking, drinking, sleep quality, fruits/vegetable consumption, mental health, and PM_2.5_ in addition to Model 2. Potential mediation effects of sleep quality and mental health were further tested by examining changes in the effect estimates after adding them into Model 2. HR and 95% confidence interval (CI) were reported. Additionally, the COX regression based on restricted cubic splines (RCS) with 4 knots RCS was applied to explore nonlinear associations (R rcs package) [36–38].

Stratified analyses were conducted by the following factors: age (<55 vs. ≥55 years old), BMI (<25 vs. ≥25 kg/m^2^), educational attainment (<13 vs. ≥13 years), currently smoking (Yes vs. No), physical activity (<600 MET min/week vs. ≥600 MET min/week), fruit/vegetable consumption (low consumption group, defined as fruit consumption<2 pieces/day and vegetables<4 tablespoons/day vs. high consumption group), sleep quality grade (0,1,2,3,4), PM_2.5_ (<10 vs. ≥10 μg/m^3^), mental health (had NATD, major depression, and bipolar disorder vs. without these diseases). Further, potential interactions were evaluated by including interaction terms between the above-selected factors and road traffic noise in the primary model.

A sensitivity analysis was carried out to assess the impact of missing data by including all participants (N = 332,471) using multiple imputations (MI) for the missing data [39]. Another sensitivity analysis was conducted by excluding 2,807 patients with ear/vestibular disorders [40]. We also adjusted for the length of time at the current address to test the robustness of the results. All analyses were performed using Stata software version 14 (STATA Corp., TX, US) and R 4.0.5 (R Foundation for Statistical Computing Vienna, Austria).

## Results

### Characteristics of Study Participants

Among 305,969 participants included in the analysis, 53.43% were women, and the average age was 57.1 years. The average 24-hour noise level was 54.9dB(A) and the average nighttime noise level was 45.4dB(A). Statistically significant differences in general characteristics were found between participants with T2D and without T2D (*p* < 0.001), as presented in Table 1.

**TABLE 1:** Basic characteristics of the study participants in the United Kingdom biobank. (United Kingdom, 2006–2021).

	Total (*n* = 305,969)	Without T2D (*n* = 286,666)	T2D (*n* = 19,303)	*p* Value
Age, mean (SD), y	57.1 ± 8.10	57.0 ± 8.12	59.8 ± 7.28	<0.001
Gender (Women, %)	163,483 (53.43)	155,971 (54.41)	7,512 (38.92)	<0.001
White ethnicity (%)	290,813 (95.05)	273,586 (95.44)	17,227 (89.25)	<0.001
BMI (kg/m^2^)	27.32 ± 4.67	27.05 ± 4.48	31.27 ± 5.52	<0.001
MET≥600 min/week (%)	248,880 (81.34)	234,676 (81.86)	14,204 (73.58)	<0.001
Current smoker (%)	31,204 (10.20)	28,667 (10.00)	2,537 (13.14)	<0.001
Current drinker (%)	283,452 (92.64)	266,635 (93.01)	16,817 (87.12)	<0.001
Education ≥13 years (%)	174,351 (56.98)	165,009 (57.56)	9,342 (48.40)	<0.001
Fruits (pieces/day)	1.92 ± 2.61	1.92 ± 2.61	1.98 ± 2.68	<0.001
Vegetables (tablespoons/day)	4.32 ± 4.74	4.32 ± 4.71	4.27 ± 5.15	<0.001
Sleep quality^a^ (%)				<0.001
0–1	22,212 (7.26)	19,609 (6.84)	2,603 (13.48)	
2	69,744 (22.79)	64,113 (22.37)	5,631 (29.17)	
3	120,845 (39.50)	113,524 (39.60)	7,321 (37.93)	
4	93,168 (30.45)	89,420 (31.19)	3,748 (19.42)	
Mental health^b^ (%)	108,428 (35.44)	101,210 (35.31)	7,218 (37.39)	<0.001
PM2.5≥10 µg/m^3^ (%)	142,330 (46.52)	132,303(46.15)	10,027 (51.95)	<0.001
Road traffic noise (Lden)	54.9 (53.5–57.0)	54.9 (53.5–57.0)	55.0 (53.5–57.1)	<0.001
Road traffic noise (Lnight)	45.4 (44.0, 47.5)	45.4 (44.0–47.5)	45.5 (44.1–47.7)	<0.001

a Sleeping quality was evaluated by a healthy sleep score (0–4) calculated using sleep duration, insomnia, snoring, and nap during the day.

b Mental health had symptoms of nerves, anxiety, nervousness or depression, major depression, and bipolar disorder vs. without these diseases.

BMI, body mass index; MET, metabolic equivalent; PM_2.5_, particulate matter less than or equal to 2.5 μg/m.

### Associations Between Road Traffic Noise and T2D

During a median follow-up period of 11.9 years, a total of 19,303 participants (6.3%) newly diagnosed with T2D. After adjusting for age, gender, ethnicity, educational attainment, physical activity, and PM_2.5_, participants in the highest quartile of road traffic noise level had a higher risk of T2D compared to those in the lowest quartile (HR = 1.03, 95% CI: 0.99–1.07). For per 10 dB(A) increase in 24-hour road traffic noise, the risk of T2D increased by 4% (95% CI: 1.01–1.07, *p* < 0.001) (Table 2). With additional adjustments for smoking, drinking, sleep quality, fruits/vegetable consumption, and mental health, the associations were attenuated (Table 2). Similar associations were observed for nighttime road traffic noise (Supplementary Table S1).

**TABLE 2:** Hazard ratios of type 2 diabetes by road traffic noise among 305,967 United Kingdom Biobank participants. (United Kingdom, 2006–2021).

Road traffic noise	Model 1	Model 2	Model 2 + PM_2.5_	Model 2 + sleep quality	Model 2 + mental health	Model 3
	HR (95%CI)	HR (95%CI)	HR (95%CI)	HR (95%CI)	HR (95%CI)	HR (95%CI)
Lden per 10 dB	1.09 (1.06, 1.13)	1.09 (1.05, 1.12)	1.04 (1.01, 1.07)	1.01 (1.00, 1.01)	1.01 (1.00, 1.01)	1.03 (1.00, 1.06)
Quintile 1 (<53.5)	References	References	References	References	References	References
Quintile 2 (53.5–54.9)	1.02 (0.98, 1.06)	1.01 (0.97, 1.05)	1.00 (0.96, 1.04)	1.01 (0.97, 1.05)	1.01 (0.97, 1.05)	1.00 (0.96, 1.04)
Quintile 3 (54.9–57.0)	1.03 (0.98, 1.07)	1.03 (0.99, 1.08)	1.00 (0.96, 1.04)	1.03 (0.99, 1.08)	1.03 (0.99, 1.07)	1.00 (0.96, 1.04)
Quintile 4 (≥57.0)	1.08 (1.03, 1.12)	1.08 (1.04, 1.13)	1.03 (0.99, 1.07)	1.08 (1.04, 1.12)	1.08 (1.04, 1.13)	1.02 (0.98, 1.06)

Model 1: unadjusted.

Model 2: adjusted for age, gender, ethnicity, physical activity, educational attainment.

Model 2+ PM_2.5._

Model 2 + sleep quality.

Model 2 + mental health.

Model 3: adjusted for all the covariates in Model 2 + smoking, drinking, body mass index, sleep quality, fruits/vegetable consumption, mental health, and PM_2.5._ T2D, Type 2 diabetes; HR, hazard ratio; CI, confidence interval; PM_2.5_, particulate matter less than or equal to 2.5 μg/m.

### Subgroup Analysis of the Associations Between Road Traffic Noise and T2D

**FIGURE 1:**
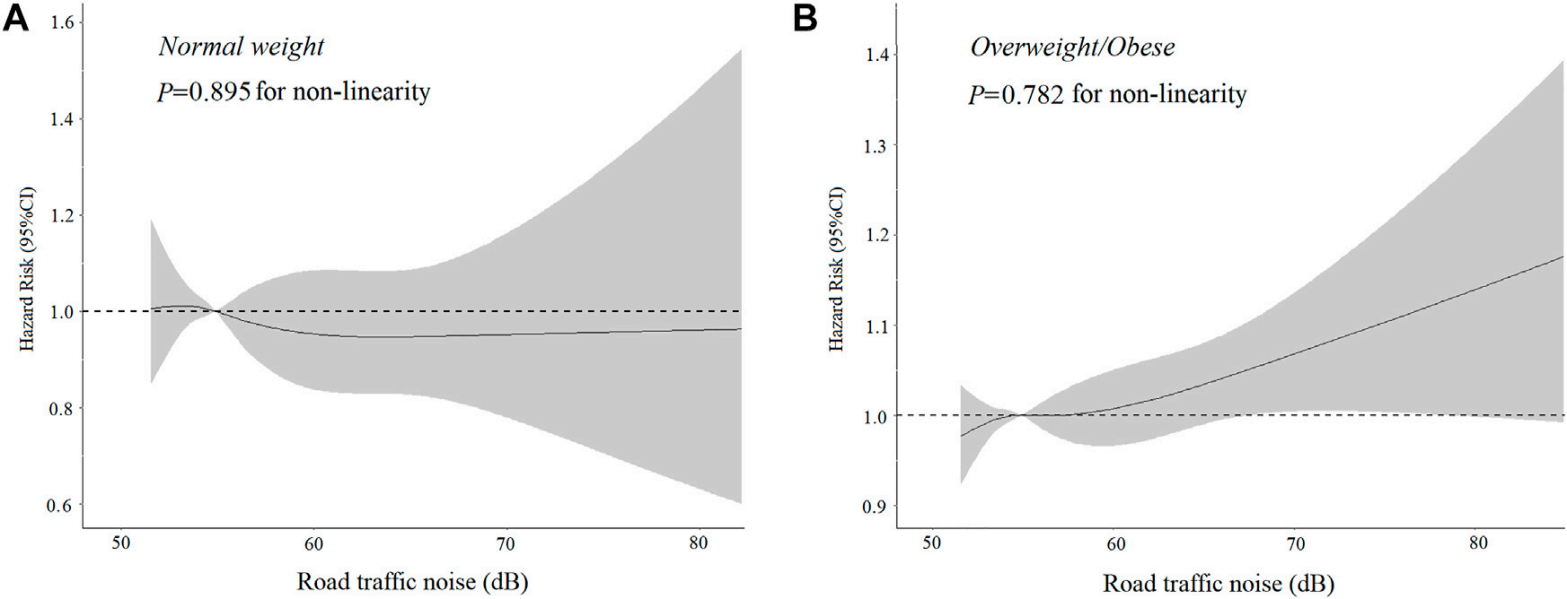
Dose-response association of road traffic noise with **(A)** Type 2 diabetes incidence among normal-weight individuals. **(B)** Type 2 diabetes incidence among overweight/obese individuals (United Kingdom, 2006–2021). *Adjusted for age, gender, ethnicity, physical activity, educational attainment, and particulate matter less than or equal to 2.5 μg/m.

There was a significant interaction between BMI and road traffic noise (*P* interaction <0.001) on the risk of T2D, suggesting a potential modification effect of obesity on the association between road traffic noise and the risk of T2D. The stratified analysis showed that the association of road traffic noise with T2D was stronger in overweight and obese participants (for per 10 dB(A) increase: HR = 1.04, 95% CI: 1.01–1.08), but not significant among lean ones (for per 10 dB(A) increase: HR = 0.96, 95% CI: 0.86–1.07) (Table 3). Similarly, a significant linear relationship between road traffic noise exposure and T2D risk among overweight and obese participants was observed when road traffic noise exposure was treated as a continuous variable (Figure 1).

**TABLE 3:** Associations between road traffic noise and type 2 diabetes by body mass index (United Kingdom, 2006–2021).

Road traffic noise	Model 1	Model 2	Model 2 + PM_2.5_	Model 2 + sleep quality	Model 2 + mental health	Model 3
	HR (95%CI)	HR (95%CI)	HR (95%CI)	HR (95%CI)	HR (95%CI)	HR (95%CI)
BMI<25 (*n* = 101,978)						
Lden per 10 dB	1.00 (0.90, 1.12)	0.99 (0.89, 1.11)	0.96 (0.86, 1.07)	0.99 (0.89, 1.10)	0.99 (0.89, 1.10)	0.94 (0.84, 1.05)
Quintile 1 (<53.5)	References	References	References	References	References	References
Quintile 2 (53.5–54.9)	1.07 (0.94, 1.21)	1.05 (0.92, 1.19)	1.04 (0.91, 1.18)	1.05 (0.92, 1.19)	1.05 (0.92, 1.19)	1.04 (0.91, 1.18)
Quintile 3 (54.9–57.0)	1.02 (0.90, 1.16)	1.02 (0.89, 1.16)	0.99 (0.87, 1.13)	1.02 (0.89, 1.16)	1.02 (0.89, 1.16)	0.99 (0.87, 1.13)
Quintile 4 (≥57.0)	1.00 (0.88, 1.14)	1.00 (0.88, 1.14)	0.96 (0.84, 1.10)	1.00 (0.88, 1.14)	1.00 (0.88, 1.14)	0.95 (0.83, 1.08)
BMI ≥25 (*n* = 203,989)						
Lden per 10 dB	1.10 (1.06, 1.13)	1.09 (1.05, 1.13)	1.04 (1.01, 1.08)	1.09 (1.05, 1.12)	1.09 (1.05, 1.12)	1.03 (1.00, 1.07)
Quintile 1 (<53.5)	References	References	References	References	References	References
Quintile 2 (53.5–54.9)	1.02 (0.98, 1.06)	1.01 (0.97, 1.06)	1.00 (0.96, 1.05)	1.01 (0.97, 1.06)	1.01 (0.97, 1.06)	1.00 (0.96, 1.05)
Quintile 3 (54.9–57.0)	1.03 (0.99, 1.07)	1.04 (0.99, 1.08)	1.00 (0.96, 1.04)	1.03 (0.99, 1.08)	1.03 (0.99, 1.08)	1.00 (0.96, 1.04)
Quintile 4 (≥57.0)	1.09 (1.04, 1.13)	1.09 (1.05, 1.14)	1.04 (1.00, 1.08)	1.09 (1.04, 1.13)	1.09 (1.05, 1.14)	1.03 (0.99, 1.08)

Model 1: unadjusted.

Model 2: adjusted for age, gender, ethnicity, physical activity, educational attainment.

Model 2+ PM_2.5._

Model 2 + sleep quality.

Model 2 + mental health.

Model 3: adjusted for all the covariates in Model 2 + smoking, drinking, sleep quality, fruits/vegetable consumption, mental health, and PM_2.5_.

T2D, type 2 diabetes; HR, hazard ratio; CI, confidence interval; PM_2.5_, particulate matter less than or equal to 2.5 μg/m.

The interactions between road traffic noise and other potential risk factors for T2D were also tested. Significant interactions of road traffic noise exposure with smoking, drinking, and PM_2.5_ for T2D were observed (*p* < 0.05), indicating that road traffic exposure might have a greater impact on the population who were non-smokers, current drinkers, or exposed to PM_2.5_≥10 μg/m^3^ (Supplementary Table S2).

### Sensitivity Analysis

The association of road traffic noise with T2D did not change significantly when including all participants using imputed missing data (Supplementary Table S3), excluding the ones with ear/vestibular disorder (Supplementary Table S4), or adjusted for the period of residence (Supplementary Table S5).

## Discussion

We reported a positive association between road traffic noise exposure and T2D using a large prospective population-based cohort study from UK Biobank. In addition, for the first time, we found that obesity might moderate the impact of road traffic noise on T2D, with a stronger association observed in overweight and obese individuals. Stratified analyses also indicated stronger associations among those exposed to a concentration of PM_2.5_ ≥10 μg/m^3^.

Our results are consistent with most previous prospective cohort studies, which found that exposure to road traffic noise was significantly associated with an increased risk of T2D [12–16, 18, 41, 42]. A large cohort of 914,607 participants from Toronto reported an 8% increase in diabetes risk for every 10 dB increase in road traffic noise [15]. A recent Danish National Cohort study reported that long-term exposure to road traffic noise was associated with an increased risk of diabetes [41]. Another two recent cohort studies conducted in Canada also reported a significant association between residential road traffic noise and increased risk of diabetes. However, these studies did not take important individual-level risk factors into account, such as smoking history and diet [15, 18]. Our results further confirmed that the association remained statistically significant with additional adjustment for these factors. Also, we found weak evidence that sleep quality and mental health mediate the associations of road traffic noise exposure with T2D.

Older individuals (age ≥55 years) have an increased risk of T2D due to exposure to road traffic noise. One possible explanation is that the elderly usually have poor sleep quality (Supplementary Table S6) [12, 43]. Similar to our study (Supplementary Table S7), Thacher et al. reported that individuals living in areas with higher levels of PM_2.5_ tended to show a stronger association between road traffic noise and diabetes incidence [41]. We found no risk of road traffic noise-related diabetes among current smokers, suggesting that traffic noise and smoking may share a similar pathway to T2D. In addition, we observed a stronger association between road traffic noise and T2D among current drinkers. The underlying mechanisms of these interactions need to be further studied.

Previous studies have revealed several potential mechanisms linking road traffic noise exposure to an increased risk of T2D. First, road traffic noise induces oxidative response as a stressor, which increases the level of glucocorticoid, insulin resistance, and subsequent inflammation of visceral adipose tissue [44, 45]. Second, noise exposure increases systemic inflammatory reaction, leading to dysfunction of pancreatic β cells, which in turn results in insulin resistance [46, 47]. Third, nighttime noise exposure affects sleep disturbances [20, 48] (Supplementary Table S8), which are associated with changes in glucose and appetite regulation [14, 49], as well as inflammation and metabolism [50, 51], all of which increase the risk of T2D. Lastly, noise may alter the composition and function of the human gut microbiome, then lead to persistent abnormalities in blood sugar regulation [52]. Mechanistic exploration is out of the scope of this research, and additional studies are required to examine other potential underlying pathways.

An interesting finding of this study is that obesity significantly altered the effect of road traffic noise exposure on T2D, suggesting that road traffic noise and obesity may jointly affect T2D, but the mechanism is unclear. Adverse effects of noise exposure on health are more apparent among overweight and obese individuals, which was also observed in other diseases such as hypertension [19], dementia, and cognitive impairment [20]. Noise exposure activates the hypothalamic-pituitary-adrenal axis (HPA) to induce glucocorticoid overproduction [50, 53–55], further inhibiting insulin secretion [56]. Studies have shown that obesity is associated with the overactivity of the HPA axis [57]. In addition, several inflammatory cytokines associated with obesity, such as adiponectin and leptin, may aggravate the inflammatory process induced by noise exposure [58–61]. The association between cortisol produced by noise exposure as a stressor and obesity may be bidirectional. Obese individuals tend to report higher perceived stress [62], promoting more cortisol production [63], which in turn may promote the accumulation of fat cells and ultimately lead to obesity [64]. More studies are required to account for the potential biological mechanisms of these associations.

The main strength of this study is that we investigate the association between road traffic noise exposure and T2D using a large prospective cohort study, with an average follow-up of 11.9 years and over 300,000 participants. This study also has several limitations. First, the observational nature of this study is not sufficient to infer causality. Second, the road traffic noise estimation model without considering such factors as time at home, room layout, the habit of a window opening, and noise sensitivity, may lead to the classification error of noise exposure. Also, the simplified model tended to overestimate noise exposure at low levels due to the assumed national traffic flow baseline value but to underestimate exposure for those heavily trafficked minor roads [65], thus leading to a narrow range of road traffic noise exposure. Further studies measuring precise individual road traffic noise exposure, such as measuring indoor noise or using more sophisticated noise, are recommended. Third, the results may not be generalizable to other populations. Finally, we were not able to model the changes in road traffic noise over the follow-up period due to data limitation. However, the main results were robust in a sensitivity analysis adjusting the period of residence and found similar results.

### Conclusion

In a nutshell, road traffic noise exposure is associated with a increased risk of T2D, and the association may be modified by obesity. More research is required to reveal the underlying biological mechanism.
