# Supplementary material for: Road Traffic Noise, Obesity, and the Risk of Incident Type 2 Diabetes: A Cohort Study in UK Biobank
Source: Int J Public Health. 2022 Oct 12;67:1605256. doi: 10.3389/ijph.2022.1605256 (PMC9596764; doi:10.3389/ijph.2022.1605256)
Supplement: Supplementary file 1 [file DataSheet2.docx]

**Supplementary methods**

**Assessment of sleep**

To assess sleep quality, we used UKB’s touchscreen questionnaire on sleep at baseline. Data fields of interest were (1) UKB sleep duration (Field ID:1160; About how many hours sleep do you get in every 24 hours? (please include naps)), (2) Nap during day sleep (Field ID: 1190; Do you have a nap during the day?)). (3) Sleeplessness/ insomnia sleep (Field ID: 1200; Do you have trouble falling asleep at night or do you wake up in the middle of the night?; coded as never/rarely, sometimes, usually, prefer not to answer), and (4) Snoring (Field ID: 1210; Does your partner or a close relative or friend complain about your snoring?; coded as yes, no, do not know, prefer not to answer).

**Assessment of mental health**

For mental health assessment, data fields of interest included (1) UKB Mental disorders (Field ID:2090; Have you ever seen a general practitioner (GP) for nerves, anxiety, tension or depression?), (2) UKB Mental disorders (Field ID:2100; Have you ever seen a psychiatrist for nerves. anxiety, tension or depression?), (3) UKB Bipolar and major depression status (Field ID:20126; coded as 0: No Bipolar or Depression, 1: Bipolar I Disorder, 2: Bipolar II Disorder, 3: Probable Recurrent major depression (severe), 4: Probable Recurrent major depression (moderate), 5: Single Probable major depression episode).
