# Supplementary material for: Road Traffic Noise, Obesity, and the Risk of Incident Type 2 Diabetes: A Cohort Study in UK Biobank
Source: Int J Public Health. 2022 Oct 12;67:1605256. doi: 10.3389/ijph.2022.1605256 (PMC9596764; doi:10.3389/ijph.2022.1605256)
Supplement: Supplementary file 2 [file DataSheet1.docx]

| **Supplementary Table 1. Associations between night-time traffic noise and type 2 diabetes (United Kingdom, 2006-2021).** | | | | | | |
| --- | --- | --- | --- | --- | --- | --- |
| Road traffic noise | Model 1 | | Model 2 | | Model 3 | |
|  | HR (95% CI) | *P* value | HR (95% CI) | *P* value | HR (95% CI) | *P* value |
| Lden per 10dB | 1.09 (1.06,1.13) | <0.001 | 1.09 (1.05,1.12) | <0.001 | 1.03 (1.00,1.06) | 0.078 |
| Quartile 1 (<53.5) | Reference |  | Reference |  | Reference |  |
| Quartile 2 (53.5-54.9) | 1.02 (0.98,1.06) | 0.422 | 1.01 (0.97,1.05) | 0.643 | 1.00 (0.96,1.04) | 0.879 |
| Quartile 3 (54.9-57.0) | 1.03 (0.99,1.07) | 0.220 | 1.03 (0.99,1.07) | 0.126 | 1.00 (0.96,1.04) | 0.976 |
| Quartile 4 (≥57.0) | 1.08 (1.03,1.12) | <0.001 | 1.08 (1.04,1.12) | <0.001 | 1.02 (0.98,1.07) | 0.260 |
| Model 1: unadjusted; Model 2: adjusted for age, gender, ethnicity, physical activity, educational attainment; Model 3: adjusted for all the covariates in Model 2 + smoking, drinking, sleep quality, fruits/vegetable consumption, mental health, and particulate matter less than or equal to 2.5µg/m. HR=hazard ratio; CI=confidence interval. | | | | | | |

| **Supplementary Table 2. Associations of road traffic noise with type 2 diabetes (stratified analysis) (United Kingdom, 2006-2021).** | | | |
| --- | --- | --- | --- |
| Subgroups, Noise (Lden, dB(A)) | N cases | HR (95% CI) | *P* for interaction |
| Sex |  |  | 0.206 |
| Male | 11,789 | 1.03 (0.99,1.08) |  |
| Female | 7,512 | 1.05 (0.99,1.10) |  |
| Age, years |  |  | 0.075 |
| Age<55 | 4,777 | 1.00 (0.94,1.07) |  |
| Age≥55 | 14,526 | 1.05 (1.01,1.09) |  |
| BMI, kg/m2 |  |  | <0.001 |
| BMI<25 | 1,874 | 0.96 (0.86,1.07) |  |
| BMI≥25 | 17,427 | 1.04 (1.01,1.08) |  |
| Educational attainment |  |  | 0.146 |
| ≥13 years | 9,341 | 1.06 (1.02,1.11) |  |
| <13 years | 9,960 | 1.02 (0.97,1.06) |  |
| Current smokers |  |  | 0.009 |
| Yes | 2,536 | 1.01 (0.93,1.11) |  |
| No | 16,765 | 1.04 (1.00,1.18) |  |
| Current drinkers |  |  | 0.021 |
| Yes | 16,815 | 1.05 (1.02,1.09) |  |
| No | 2,486 | 0.94 (0.86,1.03) |  |
| Physical activity |  |  | 0.843 |
| ≥MET 600 min/w | 14,202 | 1.03 (0.99,1.07) |  |
| <MET 600 min/w | 5,099 | 1.06 (0.99,1.13) |  |
| Fruits/Vegs consumption* |  |  | 0.977 |
| High | 15,798 | 1.04 (1.00,1.08) |  |
| Low | 3,503 | 1.04 (0.97,1.12) |  |
| sleep quality |  |  | 0.918 |
| 0-1 | 2,602 | 1.09 (1.00,1.18) |  |
| 2 | 5,630 | 1.01 (0.95,1.07) |  |
| 3 | 7,321 | 1.03 (0.98,1.09) |  |
| 4 | 3,748 | 1.06 (0.98,1.14) |  |
| Mental health |  |  | 0.660 |
| Yes | 7,212 | 1.03 (1.97,1.08) |  |
| No | 12,085 | 1.05 (1.00,1.09) |  |
| PM_2.5_, µg/m3 |  |  | 0.001 |
| <10 | 9,274 | 1.01 (0.95,1.08) |  |
| ≥10 | 10,027 | 1.05 (1.01,1.09) |  |
| BMI=body mass index; MET= Metabolic equivalent; PM_2.5_=Particulate matter less than or equal to 2.5µg/m3; HR=Hazard ratio; CI=Confidence interval. *Low consumption group was defined as fruit consumption<2 pieces/day and vegetables <4 tablespoons/day, and high consumption group was fruit consumption≥2 pieces/day or vegetables ≥4 tablespoons/day. Adjusted for age, gender, ethnicity, physical activity, educational attainment, PM_2.5_. | | | |

| **Supplementary Table 3. Hazard risks of type 2 diabetes by road traffic noise among UK Biobank participants (imputed data) (United Kingdom, 2006-2021).** | | | | | | |
| --- | --- | --- | --- | --- | --- | --- |
| Road traffic noise | Model 1 | | Model 2 | | Model 3 | |
|  | HR (95% CI) | *P* value | HR (95% CI) | *P* value | HR (95% CI) | *P* value |
| Lden per 10dB | 1.09 (1.06,1.12) | <0.001 | 1.11 (1.08,1.14) | <0.001 | 1.01 (0.99,1.05) | 0.320 |
| Quartile 1 (<53.5) | Reference |  | Reference |  | Reference |  |
| Quartile 2 (53.5-54.9) | 1.02 (0.99,1.06) | 0.177 | 1.03 (0.99,1.06) | 0.124 | 1.01 (0.97,1.05) | 0.608 |
| Quartile 3 (54.9-57.0) | 1.05 (1.02,1.09) | 0.003 | 1.07 (1.03,1.11) | <0.001 | 1.02(0.98,1.06) | 0.258 |
| Quartile4 (≥57.0) | 1.09 (1.05,1.13) | <0.001 | 1.11 (1.08,1.15) | <0.001 | 1.02 (0.99,1.06) | 0.227 |
| Model 1: unadjusted; Model 2: adjusted for age, gender, ethnicity, physical activity, educational attainment; Model 3: adjusted for all the covariates in Model 2 + smoking, drinking, sleep quality, fruits/vegetable consumption, mental health, and particulate matter less than or equal to 2.5µg/m. HR=hazard ratio; CI=confidence interval. | | | | | | |

| **Supplementary Table 4. Associations between traffic noise and type 2 diabetes (excluding participants with ear/ vestibular disorder) (United Kingdom, 2006-2021).** | | | | | | |
| --- | --- | --- | --- | --- | --- | --- |
| Road traffic noise | Model 1 | | Model 2 | | Model 3 | |
|  | HR (95% CI) | *P* value | HR (95% CI) | *P* value | HR (95% CI) | *P* value |
| Lden per 10dB | 1.10 (1.06,1.13) | <0.001 | 1.09 (1.06,1.12) | <0.001 | 1.03 (1.00,1.06) | 0.073 |
| Quartile 1 (<53.5) | Reference |  | Reference |  | Reference |  |
| Quartile 2 (53.5-54.9) | 1.02 (0.98,1.06) | 0.376 | 1.01 (0.97,1.05) | 0.537 | 1.00 (0.96,1.05) | 0. 844 |
| Quartile 3 (54.9-57.0) | 1.03 (0.99,1.07) | 0.185 | 1.04 (0.99,1.08) | 0.078 | 1.00 (0.96,1.04) | 0.953 |
| Quartile 4 (≥57.0) | 1.08 (1.04,1.12) | <0.001 | 1.08 (1.04,1.13) | <0.001 | 1.02 (0.98,1.07) | 0.270 |
| Model 1: unadjusted; Model 2: adjusted for age, gender, ethnicity, physical activity, educational attainment; Model 3: adjusted for all the covariates in Model 2 + smoking, drinking, sleep quality, fruits/vegetable consumption, mental health, and particulate matter less than or equal to 2.5µg/m. HR=hazard ratio; CI=confidence interval. | | | | | | |

| **Supplementary Table 5. Associations between traffic noise and type 2 diabetes (United Kingdom, 2006-2021).** | | | | | | |
| --- | --- | --- | --- | --- | --- | --- |
| Road traffic noise | Model 1 | | Model 2 | | Model 3 | |
|  | HR (95% CI) | *P* value | HR (95% CI) | *P* value | HR (95% CI) | *P* value |
| Lden per 10dB | 1.09 (1.06,1.13) | <0.001 | 1.09 (1.05,1.12) | <0.001 | 0.99 (0.96,1.04) | 0.537 |
| Quartile 1 (<53.5) | Reference |  | Reference |  | Reference |  |
| Quartile 2 (53.5-54.9) | 1.02 (0.98,1.06) | 0.419 | 1.01 (0.97,1.06) | 0.508 | 1.00 (0.96,1.04) | 0.909 |
| Quartile 3 (54.9-57.0) | 1.02 (0.98,1.07) | 0.245 | 1.04 (0.99,1.08) | 0.088 | 0.99 (0.96,1.04) | 0.807 |
| Quartile 4 (≥57.0) | 1.07 (1.03,1.12) | <0.001 | 1.08 (1.04,1.13) | <0.001 | 1.02 (0.98,1.06) | 0.304 |
| Model 1: unadjusted;  Model 2: adjusted for age, gender, ethnicity, physical activity, educational attainment, length of time at current address; Model 3: adjusted for all the covariates in Model 2 + smoking, drinking, sleep quality, fruits/vegetable consumption, mental health, particulate matter less than or equal to 2.5µg/m, and length of time at current address. HR=hazard ratio; CI=confidence interval. | | | | | | |

| **Supplemental Table 6. Basic characteristics of the study participants in the United Kingdom biobank. (n =305,969), stratified by age. (United Kingdom, 2006-2021).** | | | | |
| --- | --- | --- | --- | --- |
|  | Total  (n=305,969) | Age<55  (n=115,644) | Age≥55  (190,325) | P value |
| Gender (Women, %) | 163,483 (53.43) | 65,539 (56.67) | 97,944 (51.46) | <0.001 |
| White ethnicity (%) | 290,813 (95.05) | 106,511 (92.10) | 184,302 (96.84) | <0.001 |
| BMI (kg/m**2)** | 27.3±4.67 | 27.2±4.94 | 27.4±4.49 | <0.001 |
| MET≥600 min/week (%) | 248,880 (81.34) | 92,972 (80.40) | 155,908 (81.92) | <0.001 |
| Current smoker (%) | 31,204 (10.20) | 15,054 (13.02) | 16,150 (8.49) | <0.001 |
| Current drinker (%) | 283,452 (92.64) | 107,516 (92.97) | 175,936 (92.44) | <0.001 |
| Education ≥13 years (%) | 174,351 (56.98) | 71,135 (61.51) | 103,216 (54.23) | <0.001 |
| Fruits (pieces/day) | 1.92±2.61 | 1.70±2.76 | 2.06±2.51 | <0.001 |
| Vegetables (tablespoons/day) | 4.32±4.74 | 4.04±5.01 | 4.48±4.56 | <0.001 |
| Sleep quality^a^ (%) |  |  |  | <0.001 |
| 0-1 | 22,212 (7.26) | 7,062 (6.11) | 15,150 (7.96) |  |
| 2 | 69,744 (22.79) | 23,851 (20.62) | 45,893 (24.11) |  |
| 3 | 120,845 (39.50) | 93,168 (30.45) | 39,928 (40.02) |  |
| 4 | 93,168 (30.45) | 40,051 (34.63) | 53,117 (27.91) |  |
| Mental health^b^ (%) | 108,428 (35.44) | 43,450 (37.57) | 64,978 (34.14) | <0.001 |
| PM2.5≥10µg/m3 (%) | 142,330 (46.52) | 58,006 (50.16) | 84,324 (44.31) | <0.001 |
| Road traffic noise (Lden) | 56.0 ±4.27 | 56.2 ±4.34 | 56.0 ±4.22 | <0.001 |
| Road traffic noise (Lnight) | 46.6±4.27 | 46.7±4.34 | 46.5±4.22 | <0.001 |
| Ear disorder (%) | 2,807 (0.92) | 836 (0.72) | 1,971 (1.04) | 0.025 |
| BMI=body mass index; MET=metabolic equivalent; PM_2.5_=particulate matter less than or equal to 2.5µg/m | | | | |
| a: Sleeping quality was evaluated by a healthy sleep score (0-4) calculated using sleep duration, insomnia, snoring, and nap during the day. | | | | |
| b: mental health had symptoms of nerves, anxiety, tension or depression, major depression, and bipolar disorder vs. without these diseases. | | | | |

| **Supplemental Table 7. Basic characteristics of the study participants in the United Kingdom biobank. (n =305,969), stratified by median particulate matter less than or equal to 2.5µg/m (United Kingdom, 2006-2021).** | | | | |
| --- | --- | --- | --- | --- |
|  | Total  (n=305,969) | PM_2.5_<10 (n=163639) | PM_2.5_≥10 (n=142,330) | P value |
| Age, mean (SD), y | 57.1±8.10 | 57.6±7.97 | 56.6±8.21 | <0.001 |
| Gender (Women, %) | 163,483 (53.43) | 87,249 (53.32) | 76,234 (53.56) | 0.178 |
| White ethnicity (%) | 290,813 (95.05) | 158,295 (96.73) | 132,51 (93.11) | <0.001 |
| BMI (kg/m**2)** | 27.3±4.67 | 27.2±4.51 | 27.5±4.84 | <0.001 |
| MET≥600 min/week (%) | 248,880 (81.34) | 133,556 (81.62) | 115,324 (81.03) | <0.001 |
| Current smoker (%) | 31,204 (10.20) | 13,712 (8.38) | 17,492 (12.29) | <0.001 |
| Current drinker (%) | 283,452 (92.64) | 153,23 (93.64) | 130,220 (91.49) | <0.001 |
| Education ≥13 years (%) | 174,351 (56.98) | 95,062 (58.09) | 79,289 (55.71) | <0.001 |
| Fruits (pieces/day) | 1.92±2.61 | 1.93±2.57 | 1.92±2.66 | <0.001 |
| Vegetables (tablespoons/day) | 4.32±4.74 | 4.35±4.56 | 4.28±4.94 | <0.001 |
| Sleep quality^a^ (%) |  |  |  | <0.001 |
| 0-1 | 22,212 (7.26) | 11,058 (6.76) | 11,154 (7.84) |  |
| 2 | 69,744 (22.79) | 36,278 (22.17) | 33,466 (23.51) |  |
| 3 | 120,845 (39.50) | 65,144 (39.81) | 55,701 (39.14) |  |
| 4 | 93,168 (30.45) | 51,159 (31.26) | 42,009 (29.52) |  |
| Mental health^b^ (%) | 108,428 (35.44) | 55,621 (33.99) | 52,807 (37.10) | <0.001 |
| Road traffic noise (Lden) | 56.0 ±4.27 | 55.2 ±3.39 | 57.0 ±4.91 | <0.001 |
| Road traffic noise (Lnight) | 46.6±4.27 | 45.7±3.39 | 47.5±4.91 | <0.001 |
| Ear disorder (%) | 2,807 (0.92) | 1,413 (0.86) | 1,394 (0.98) | 0.001 |
| BMI=body mass index; MET=metabolic equivalent; PM_2.5_=particulate matter less than or equal to 2.5µg/m. | | | | |
| a: Sleeping quality was evaluated by a healthy sleep score (0-4) calculated using sleep duration, insomnia, snoring, and nap during the day. | | | | |
| b: mental health had symptoms of nerves, anxiety, tension or depression, major depression, and bipolar disorder vs. without these diseases. | | | | |

| **Supplemental Table 8. Basic characteristics of the study participants in the United Kingdom biobank. (n =305,969), stratified by body mass index. (United Kingdom, 2006-2021).** | | | | |
| --- | --- | --- | --- | --- |
|  | Total  (n=305,969) | BMI<25  (n=101,978) | BMI ≥25  (n=203,991) | P value |
| Age, mean (SD), y | 57.1±8.10 | 56.4±8.26 | 57.5±7.98 | <0.001 |
| Gender (Women, %) | 163,483 (53.43) | 66,149 (64.87) | 97,334 (47.71) | <0.001 |
| White ethnicity (%) | 290,813 (95.05) | 97,268 (95.38) | 193,545 (94.88) | <0.001 |
| MET≥600 min/week (%) | 248,880 (81.34) | 87,068 (85.38) | 161,812 (79.32) | <0.001 |
| Current smoker (%) | 31,204 (10.20) | 11,201 (10.98) | 20,003 (9.81) | <0.001 |
| Current drinker (%) | 283,452 (92.64) | 94,860 (93.02) | 188,592 (92.45) | <0.001 |
| Education ≥13 years (%) | 174,351 (56.98) | 63,771 (62.53) | 110,580 (54.21) | <0.001 |
| Fruits (pieces/day) | 1.92±2.61 | 1.98±2.56 | 1.90±2.64 | <0.001 |
| Vegetables (tablespoons/day) | 4.32±4.74 | 4.35±4.64 | 4.30±4.79 | <0.001 |
| Sleep quality^a^ (%) |  |  |  | <0.001 |
| 0-1 | 22,212 (7.26) | 4,823 (4.73) | 17,389 (8.52) |  |
| 2 | 69,744 (22.79) | 19,307 (18.93) | 50,437 (24.73) |  |
| 3 | 120,845 (39.50) | 37,920 (37.18) | 82,925 (40.65) |  |
| 4 | 93,168 (30.45) | 39,928 (39.15) | 53,240 (26.10) |  |
| Mental health^b^ (%) | 108,428 (35.44) | 35,750 (35.06) | 72,678 (35.63) | 0.002 |
| PM2.5≥10µg/m3 (%) | 142,330 (46.52) | 46,788 (45.88) | 95,542 (46.84) | <0.001 |
| Road traffic noise (Lden) | 56.0 ±4.27 | 56.0 ±4.20 | 56.1 ±4.30 | <0.001 |
| Road traffic noise (Lnight) | 46.6±4.27 | 46.5±4.20 | 46.6±4.30 | <0.001 |
| Ear disorder (%) | 2,807 (0.92) | 880 (0.86) | 1,927 (0.94) | 0.025 |
| BMI=body mass index; MET=metabolic equivalent; PM_2.5_=particulate matter less than or equal to 2.5µg/m. | | | | |
| a: Sleeping quality was evaluated by a healthy sleep score (0-4) calculated using sleep duration, insomnia, snoring, and nap during the day. | | | | |
| b: mental health had symptoms of nerves, anxiety, tension or depression, major depression, and bipolar disorder vs. without these diseases. | | | | |


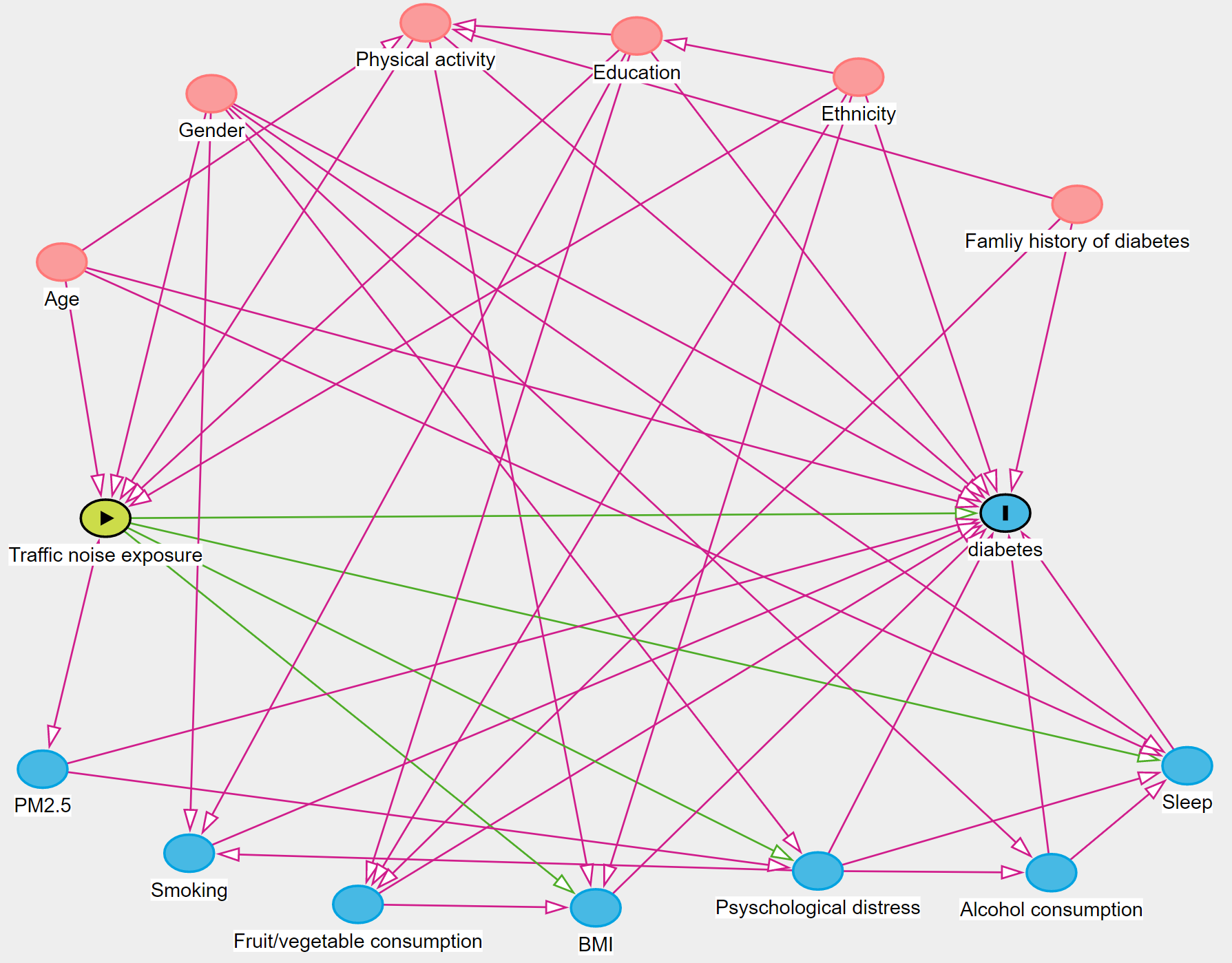


**Supplemental Figure 1. The directed acyclic causality diagram for inferring the association between road traffic noise and type 2 diabetes. (United Kingdom, 2006-2021).**
